# Mucinous cystadenocarcinoma of the appendix in which contrast-enhanced ultrasonography was useful for assessing blood flow in a focal nodular lesion in the tumor cavity: A case report

**DOI:** 10.3892/etm.2013.1094

**Published:** 2013-04-30

**Authors:** NORITAKA WAKUI, MITSURU FUJITA, YOSHIYA YAMAUCHI, YUKI TAKEDA, NOBUO UEKI, TAKAFUMI OTSUKA, NOBUYUKI OBA, SHUTA NISHINAKAGAWA, TOSHIKO TAKEZOE, JUNKO HIROYOSHI, YOSHIHARU KONO, SEIICHIRO KATAHIRA, MASAMI MINAGAWA, YASUSHI TAKEDA, SAORI SHIONO, TATSUYA KOJIMA

**Affiliations:** 1 Departments of Internal Gastroenterology and Hepatology, Tokyo Rosai Hospital, Tokyo 143-0013, Japan; 2 Surgery, Tokyo Rosai Hospital, Tokyo 143-0013, Japan; 3 Pathology, Tokyo Rosai Hospital, Tokyo 143-0013, Japan

**Keywords:** appendix, mucocele, mucinous cystadenocarcinoma, ultrasound, contrast-enhanced ultrasonography, sonazoid

## Abstract

A 63-year-old woman was admitted to hospital with pain in the right lower quadrant. Abdominal computed tomography (CT) revealed a 60-mm cystic mass at a site corresponding to the appendix. The mass wall on the appendicular ostium was thickened and enhanced by contrast, while calcification was observed in the mass wall on the appendicular tip. No projection was observed in the mass cavity. On abdominal ultrasonography (US), the mass wall on the appendicular ostium was thickened and projections were observed at two sites in the mass cavity. On contrast-enhanced US (CEUS), only one of these projections was enhanced. Based on the thickened and contrast-enhanced wall of the mass on the appendicular ostium on CT and US, as well as the contrast enhancement of a projection on US, the mass was diagnosed as mucinous cystadenocarcinoma of the appendix. Ileocecal resection was subsequently performed on day 10. A detailed examination of the surgical specimen revealed carcinoma cells in the mass wall on the appendicular ostium. The contrast-enhanced projection was identified as granulation tissue that had grown to come into contact with the tumor, while the non-contrast-enhanced projection was identified as solidified mucus. US enabled successful visualization of projections in the mass cavity that were not visible on abdominal CT. CEUS also proved useful for assessing blood flow in these projections.

## Introduction

Mucocele of the appendix is a cystoid extension of the appendix resulting from mucus accumulation in the appendix cavity (1). It is also a rare pathology of the appendix without characteristic clinical symptoms (2,3). Although mucocele of the appendix is often discovered following surgery due to the difficulty in obtaining a preoperative diagnosis, recent advances in imaging technology have led to an improved preoperative diagnostic rate. Kim *et al* (4) performed a detailed examination of mucocele of the appendix in 17 patients by computed tomography (CT) and ultrasonography (US) and suggested that the presence of a focal nodular lesion in the tumor cavity is an important predictor of malignancy.

The current report describes a case of mucinous cystadenocarcinoma of the appendix in which contrast-enhanced US (CEUS) was useful for the detailed assessment of blood flow in projections in the mass cavity. The radiographic change was observed after 1 year and 7 months. Written informed consent was obtained from the patient.

## Case report

**Figure 1.**
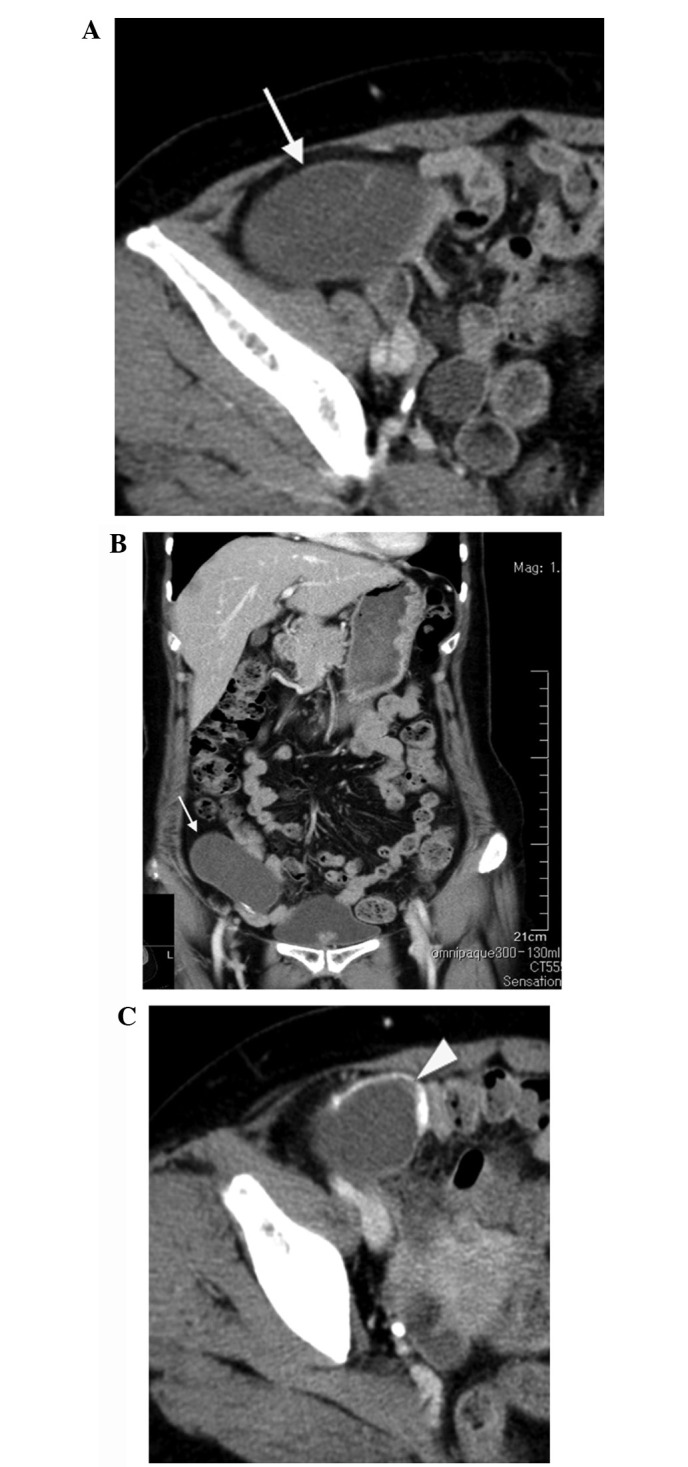
Abdominal computed tomography (CT) scans obtained in January 2011. Axial view (A) and coronal view (B) showing a 60-mm cystic mass at the site corresponding to the appendix, with no thickening or contrast enhancement in its wall. No projection was visible in the mass cavity (arrow). Another axial view (C) demonstrated calcification of the mass wall on the appendicular tip (arrowhead).

A 63-year-old female was admitted to Tokyo Rosai Hospital with discomfort in the right lower quadrant, which the patient had being experiencing since approximately January 2011. Physical examination revealed no tenderness; however, a palpable, fist-sized mass in the right lower quadrant prompted abdominal CT, which revealed a 60-mm cystic mass at the site corresponding to the appendix with calcification in its wall on the appendicular tip. With no thickening or contrast enhancement in the entire wall of the mass, cystadenoma, as opposed to carcinoma was suspected (Fig. 1). Since the possibility of carcinoma could not be ruled out, surgical removal of the mass was recommended; however, the patient refused surgery and was placed on a careful outpatient follow-up program.

Three months later, the patient returned to the hospital. Abdominal CT revealed no changes compared with the previous examination. The patient was advised to return to the outpatient clinic in 3 months; however, the patient did not return. Later, the patient presented with persistent pain in the right lower quadrant, which the patient had experienced since August 2012. On examination, a mass was felt in the right lower quadrant that resembled the one felt previously and tenderness was experienced at the same site. The patient was then admitted for workup. The patient had no history of alcohol or smoking and the prior medical history included surgery for internal hemorrhoids at the age of 59 years. No signifant family history was reported and no oral medication was being used. On admission, the patient had clear sensorium and a blood pressure of 123/73 mmHg, a pulse rate of 60 beats/min (non-arrhythmic) and a body temperature of 37.5°C. The palpebral conjunctiva was not anemic and no yellow discoloration of the bulbar conjunctiva was observed. Heart and breath sounds were noted to be clear. The abdomen was flat and soft with a palpable fist-sized mass present in the right lower quadrant. The mass was slightly hard and minimally movable with tenderness; however, no rebound tenderness or muscular rigidity was apparent. The liver and spleen were impalpable. Hematological examination on admission revealed mild anemia (hemoglobin, 11.4 g/dl) and increased inflammatory reaction (C-reactive protein, 6.5 mg/dl). No increase in the levels of tumor markers was observed (Table I).

**Figure 2.**
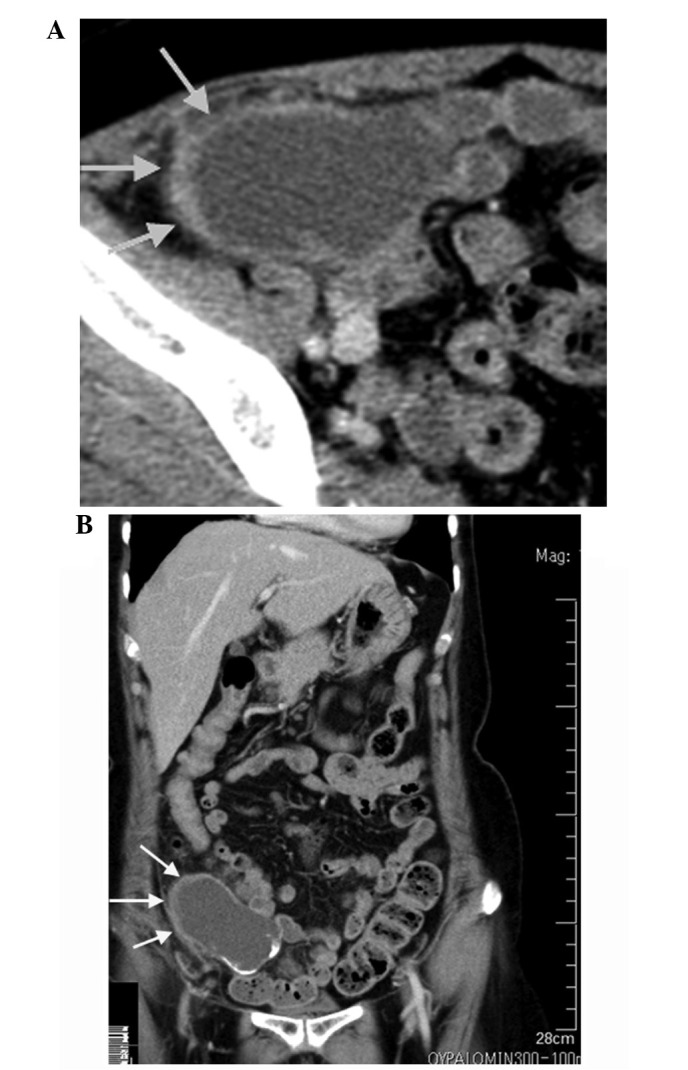
Abdominal computed tomography (CT) scans obtained in August 2012. Axial view (A) and coronal view (B) show no change in the size of the cystic mass at the site of the appendix. Thickening of the mass wall on the appendicular ostium was visible, with contrast enhancement at the corresponding site (arrow). No projection was visible in the mass cavity.

**Figure 3.**
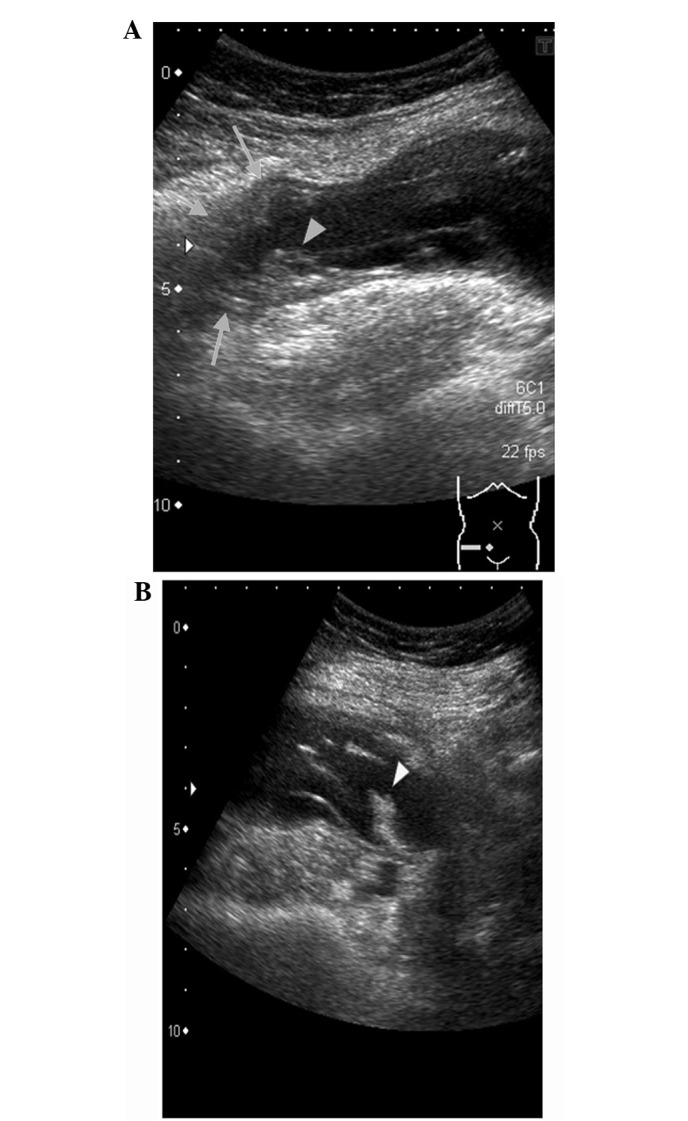
Abdominal ultrasonograms obtained in August 2012. An anechoic mass with a partly layered echo pattern was visible from the appendicular ostium (A) and from the appendicular tip (B). The mass wall on the appendicular ostium was thickened (A, arrow), with a 13-mm projection protruding toward the cavity from part of the wall. Another 9-mm projection was visible on the appendicular tip (B, arrowhead).

Abdominal CT on day 2 revealed no change in the size of the existing cystic mass from the previous CT scan performed in January 2011; however, it revealed thickening of the mass wall on the appendicular ostium and contrast enhancement at the corresponding site. No projection was observed in the mass cavity (Fig. 2). On abdominal US on day 2, the mass was anechoic overall and demonstrated a partly layered echo pattern. The mass wall on the appendicular ostium was thickened with a 13-mm projection protruding toward the cavity from part of the wall. Another 9-mm projection was also observed in the appendicular tip (Fig. 3).

**Figure 4.**
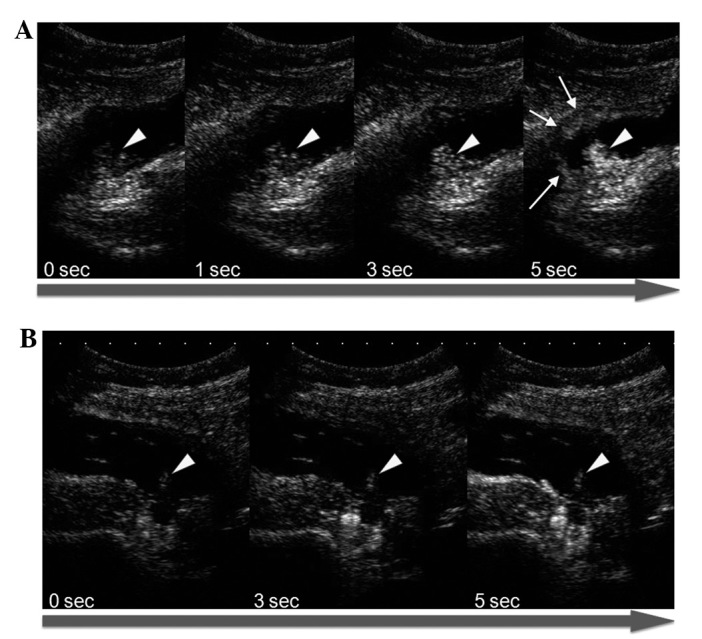
Abdominal contrast-enhanced ultrasonograms obtained in August 2012. A mass was visible from (A) the appendicular ostium and (B) from the appendicular tip. The mass wall (A, arrow) and the projection (A, arrowhead) on the appendicular ostium were enhanced within 5 sec of contrast agent arrival, whereas no enhancement was visible in the projection on the appendicular tip (B, arrow head).

CEUS was then performed to assess blood flow using a Toshiba SSA-790A US system (Aplio XG; Toshiba Medical Systems, Otawara, Japan) with a 3.75-MHz convex probe (PVT-375BT). Imaging was performed with a mechanical index of 0.21 and the focus was adjusted to the depth of the mass. After imaging conditions were set, Sonazoid (perfluorobutane; GE Healthcare, Oslo, Norway) was infused at the recommended dose of 0.015 ml/kg via the cubital vein. Contrast enhancement was observed in the thickened wall of the mass on the appendicular ostium and in the projection on the same side; however, not in the projection on the appendicular tip (Fig. 4).

Based on the thickened and contrast-enhanced wall of the mass on the appendicular ostium on abdominal CT and US, as well as contrast enhancement of the projection on the appendicular ostium on US, the mass was diagnosed as mucinous cystadenocarcinoma of the appendix and ileocecal resection was performed on day 10. The mass was excised with surrounding connective tissue with care taken not to break the mass.

### Gross pathological findings

**Figure 5.**
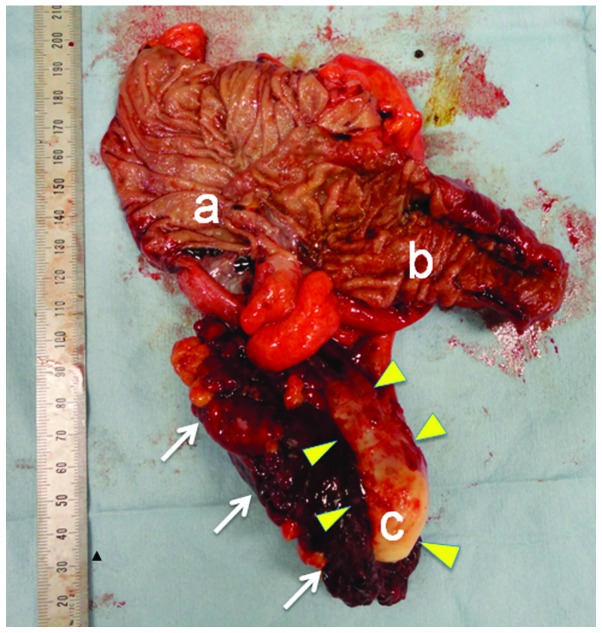
Macroscopic appearance of the excised mass. The appendix was swollen due to a 60-mm cyst with a glossy white surface (arrowhead). The mass was circumscribed by connective tissue (arrow). (a) Ileocecal region; (b) distal ileum; (c) appendix.

The appendix was swollen with a 60-mm cyst with a glossy white surface. No rupture of the mass was observed (Fig. 5).

### Histopathological findings

**Figure 6.**
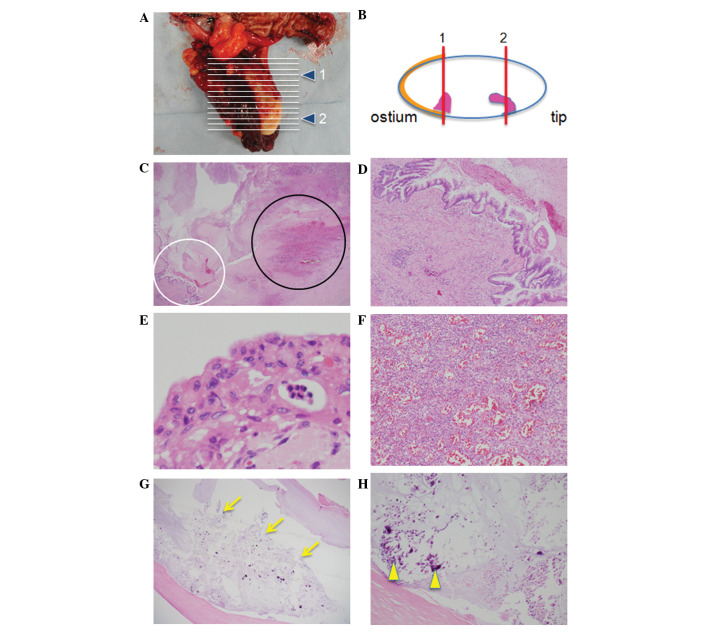
Histopathological findings of the excised mass. (A) Method for slicing the mass and (B) its schematic. Slice 1: (C) enlarged nuclei and pseudostratified cells were visible on the appendicular ostium, which led to the diagnosis of carcinoma: (white circle); hematoxylin and eosin (H&E); magnification ×1; (D) H&E; magnification, ×4; (E) H&E; magnification, ×400. In the tumor cavity [black circle in (C)]: (F) granulation tissue with proliferating capillaries was visible (H&E; magnification, ×10). Slice 2: The projection on the appendicular tip showing (G) mucus (arrow, H&E; magnification, ×1) and (H) calcification in part of the wall (arrowhead, H&E; magnification, ×100).

The mass wall was thickened on the appendicular ostium and accompanied by enlarged nuclei and pseudostratified cells, leading to the diagnosis of adenocarcinoma. The protrusion on the appendicular ostium was located inside the thickened wall and composed of granulation tissue with proliferating capillaries. The protrusion on the appendicular tip was composed of mucus, and part of the wall was calcified (Fig. 6).

## Discussion

Mucocele of the appendix is a cystoid extension of the appendix resulting from mucus accumulation in the appendix cavity that was initially described as hydrops *processus vermiformis* by Rokitansky in 1842 (1). It is a rare pathology of the appendix, accounting for 0.07-0.3% of all appendectomy cases (2,3). Mucocele of the appendix commonly affects middle-aged to older females and is often accompanied by discomfort or pain and a palpable mass in the right lower quadrant. However, symptoms are non-specific in a number of cases and 20–30% of cases are diagnosed without symptoms (5).

Kalmon and Winningingham (6) defined three factors that lead to the development of mucocele of the appendix: progressive narrowing of the valvular opening of the appendix, aseptic content and sustained mucus production. Causes of obstruction include inflammation, bending, torsion and ileocecal tumor. The most commonly used pathological classification system was developed by Higa *et al* (7), who defined the following three types and reported their respective incidence: i) focal or diffuse mucosal hyperplasia (25%); ii) mucinous cystadenoma (63%); and iii) mucinous cystadenocarcinoma (12%).

With conventional imaging techniques, findings suggestive of adenoma have been observed in a number of cases of mucinous cystadenocarcinoma of the appendix, making it difficult to distinguish precisely between the two types of lesions. In addition, since a ruptured mass may lead to pseudomyxoma peritonei (8), surgery is often performed immediately after diagnosis. However, recent advances in imaging modalities have led to improved accuracy of preoperative diagnosis of the condition (9,10), and US and CT have proved effective for diagnosing mucocele of the appendix (4,11).

On CT, the lesion is visualized as a round or oval, encapsulated, large cystic mass (12,13). Calcification of the cyst wall is highly specific to this lesion and has been shown to be a useful feature for differentiating the cyst from an abscess (4,14,15). It is considered difficult to distinguish between the two lesions based only on wall thickening and the presence of a focal nodular lesion in the tumor cavity in the cyst cavity is considered a potentially important predictor of malignancy (4). Balthazar *et al* (16) suggested that mucinous cystadenocarcinoma of the appendix is visualized on CT as an irregular, unilocular or multilocular, low-density area with infiltration into adjacent organs, which is specific compared with other types of mucocele of the appendix. In the present case, however, the lesion was visualized as a round, low-density area. This is may be due to the fact that the carcinoma arising from the appendicular ostium infiltrated only up to the mesoappendix, as confirmed pathologically.

Characteristic US findings include an anechoic or hypoechoic area in the mass (4), as well as fine punctuate, spiral or layered echo patterns (4,11,17–19). Spiral or layered echo patterns observed on US are considered to represent highly viscous mucus, which is referred to by Caspi *et al* as an ‘onion skin sign’, a finding specific to mucocele of the appendix (17). In the present case, layered echo patterns consisting primarily of an anechoic area were also observed in the mass. In addition, a thickened mass wall on the appendicular ostium and projections in the mass cavity were also observed. A previous study suggested that a definitive diagnosis of carcinoma was not made on the basis of wall thickening alone since the presence of projections in the mass cavity is an important finding that strongly suggests carcinoma (4). A detailed examination of the surgical specimen revealed that the projection on the appendicular ostium was a granulation tissue that protruded into the lumen and came into contact with the carcinoma. Although it is unclear how the granulation tissue was formed, we assume that it was a secondary reaction to proliferating carcinoma cells. Thus, a mucocele of the appendix with projection(s) in the mass cavity is likely to be solidified mucus, a mass of carcinoma cells or granulation tissue formed in response to carcinoma proliferation. The projections in the mass cavity were subjected to CEUS for assessment of blood flow. CEUS is being increasingly used as a first-line tool for detecting and characterizing hepatic liver lesions (20–25). Since its introduction to Japan in January 2007, the ultrasound contrast agent Sonazoid has been used in detailed studies on liver tumors (26–34), chronic liver disease (35–40) and other organs (41,42).

While the usefulness of color Doppler US has been suggested for determining whether a projection is mucus or a solid mass (43), CEUS provides a higher level of spatial resolution and more detailed information on blood flow and is thus used to rule out solidified mucus. We consider that CEUS is an important tool for determining the treatment strategy. With no previous study closely examining the mucocele of the appendix by CEUS, future studies should consider this modality as an important preoperative diagnostic tool for this condition.

We experienced a case of mucinous cystadenocarcinoma of the appendix in which thickening of the mass wall was observed 1 year and 7 months after the first presentation. In the present case, projections in the mass cavity, which were not visualized on abdominal CT, were successfully visualized by B-mode US. Furthermore, the use of CEUS made it possible to determine precisely whether the projections were solidified mucus or a solid tumor. These findings suggest the utility of B-mode US combined with CT for diagnostic imaging of mucocele, with CEUS being particularly useful for the assessment of blood flow in projections.

## Figures and Tables

**Table I.** Blood laboratory findings on admission.

Diagnostic blood tests	Results
Biochemistry	
CRP (mg/dl)	6.5
Na (mEq/l)	137
K (mEq/l)	4.0
Cl (mEq/l)	100
TP (g/dl)	7.9
Alb (g/dl)	4.0
T Bil (mg/dl)	0.6
D Bil (mg/dl)	0.4
AST (IU/l)	28
ALT (IU/l)	29
LDH (IU/l)	151
ALP (IU/l)	267
GGT (IU/l)	62
T Cho (mg/dl)	178
TG (mg/dl)	166
CK (IU/l)	57
BUN (mg/dl)	13
Cr (mg/dl)	0.53
BS (mg/dl)	107
HbA1c (%)	6.0
PT (%)	86
APTT (sec)	30.4
Hematology	
WBC (cells/*μ*l)	5500
RBC (cells/*μ*l)	365×10^4^
Hgb (g/dl)	11.4
Hct (%)	33.2
PLT (n/*μ*l)	23.0×10^4^
Tumor marker	
CEA (ng/ml)	2.7
CA19-9 (U/ml)	3.0
CA125 (U/ml)	13.1

CRP, C-reactive protein; Na, sodium; K, potassium; Cl, chlorine; TP, total protein; Alb, albumin; T Bil, total bilirubin; D Bil, direct bilirubin; AST, aspartate aminotransferase; ALT, alanine aminotransferase; LDH, lactate dehydrogenase; ALP, alanine phosphatase; GGT, γ-glutamyl transpeptidase; T Cho, total cholesterol; TG, triglycerides; CK, creatine kinase; BUN, blood urea nitrogen; Cr, creatinine; BS, blood sugar; HbA1c, glycosylated hemoglobin; PT, prothrombin time; APTT, activated partial thomboplastin time; WBC, white blood cells; RBC, red blood cells; Hgb, hemoglobin; Hct, hematocrit; PLT, platelet; CEA, carcinoembryonic antigen; CA, cancer antigen.

## Abbreviations:

CT: computed tomography;
US: ultrasonography
